# Italian translation and cross-cultural comparison with the Childhood Attachment and Relational Trauma Screen (CARTS)

**DOI:** 10.1080/20008198.2017.1375839

**Published:** 2017-10-10

**Authors:** A. Simonelli, C. Sacchi, L. Cantoni, M. Brown, P. Frewen

**Affiliations:** ^a^ Department of Developmental Psychology and Socialization, University of Padua, Padua, Italy; ^b^ Department of Psychology, Western University, London, Canada; ^c^ Department of Psychiatry, Western University, London, Canada; ^d^ Imaging Division, Lawson Health Research Institute, London, Canada

**Keywords:** Childhood abuse and neglect, relational trauma, attachment, psychometrics, cross-cultural, Abuso y negligencia infantil, trauma relacional, apego, psicometría, transcultural, 童年虐待和忽视, 关系创伤, 依恋, 心理测量, 跨文化, • The psychometric evaluation of an Italian version of the Childhood Attachment and Relational Trauma Screen on a group of college students. • A promising cross-cultural application of the CARTS between Italy and Canada. • The first translation of CARTS beyond the North American population.

## Abstract

**Background**: The Childhood Attachment and Relational Trauma Screen (CARTS) is a computer-administered survey designed to assess retrospectively the socio-ecological context in which instances of child abuse may have occurred. To date, studies supporting the validity of the CARTS have only been undertaken in English-speaking North American populations. Validation projects in other countries and cross-cultural comparisons are therefore warranted.

**Objective**: Develop and preliminarily evaluate the psychometric properties of an Italian version of the CARTS on college students and compare such observations to data acquired from Canadian students.

**Method**: Seventy-nine undergraduate students from the University of Padua (Italy) completed an Italian translation of the CARTS as well as measures of childhood experiences, mental health and attachment, responses to which were compared to those obtained in 288 Canadian students who completed the CARTS in English.

**Results**: Internal consistency and convergent validity with the Childhood Trauma Questionnaire and Parental Bonding Instrument were found to be acceptable for the Italian translation. Within the Italian sample, correlation analyses suggested that CARTS Mother ratings referring to attachment and abuse were associated with romantic attachment, whereas CARTS Father ratings were significantly correlated to PTSD symptoms and other symptoms of psychopathology-distress. Significant differences between Italian and Canadian students across the relationship types for the CARTS abuse and attachment scales were found, indicating that Italian students rated their mothers and fathers as simultaneously less abusive, but also less as a source of secure attachment.

**Conclusions**: The results of this preliminary study seem to suggest convergent validity of the Italian CARTS and the association between childhood attachment-related experiences and romantic attachment. Cultural variations were identified between Canadian and Italian students in both attachment and abuse scales. Future studies to investigate cross-cultural variations in the relational context of childhood abuse and in order to boost Italian CARTS psychometric features are warranted.

## Abstract (es)

**Planteamiento:** La Encuesta de Apego Infantil y Trauma Relacional (CARTS) es una encuesta administrada por ordenador diseñada para evaluar retrospectivamente el contexto socio-ecológico en el que pueden haber ocurrido casos de abuso infantil. Hasta la fecha, los estudios que apoyan la validez de la CARTS sólo se han realizado en poblaciones norteamericanas de habla inglesa. Por lo tanto, se justifican los proyectos de validación en otros países y las comparaciones interculturales.

**Objetivos:** Desarrollar y evaluar de manera preliminar las propiedades psicométricas de una versión italiana de CARTS en estudiantes universitarios y comparar dichas observaciones con datos obtenidos de estudiantes canadienses.

**Método:** Setenta y nueve estudiantes de pre-grado de la Universidad de Padua (Italia) completaron una traducción al italiano de la CARTS, así como medidas de experiencias infantiles, salud mental y apego. Las respuestas fueron comparadas con las obtenidas en 288 estudiantes canadienses que completaron la CARTS en inglés.

**Resultados:** Se encontró que la coherencia interna y la validez convergente con el Cuestionario de Trauma Infantil (Childhood Trauma Questionnaire) y el Instrumento de Vinculación Parental (Parental Bonding Instrument) eran aceptables para la traducción al italiano. Dentro de la muestra italiana, los análisis de correlación sugirieron que las puntuaciones de la CARTS-Madre que se refieren al apego y al abuso se asociaron con el apego romántico, mientras que las puntuaciones de la CARTS-Padre se correlacionaron significativamente con síntomas de TEPT y otros síntomas de trastorno psicopatológico. Se encontraron diferencias significativas entre los estudiantes italianos y canadienses entre los tipos de relación para las escalas de abuso y apego de la CARTS, lo que indica que los estudiantes italianos clasificaron a sus madres y padres simultáneamente como menos abusivos, pero también menos como fuente de apego seguro.

**Conclusiones:** Los resultados de este estudio preliminar parecen sugerir la validez convergente de la CARTS italiana y la asociación entre las experiencias relacionadas con el apego infantil y el apego romántico. Se identificaron variaciones culturales entre estudiantes canadienses e italianos en las escalas de apego y abuso. Se justifican la realización de futuros estudios para investigar las variaciones interculturales en el contexto relacional del abuso infantil y con el fin de impulsar las características psicométricas de la CARTS italiana.

## Abstract (zh)

**背景:** 童年依恋和关系创伤筛查量表（CARTS）是一个用于回溯评估可能发生儿童虐待的社会生态情景的电脑测验。迄今，仅在使用英文的北美人群中对CARTS进行过效度检验。因此还需要在其他国家进行检验，并进行跨文化的比较。

**目标：**在大学生样本中发展和初步评估意大利语版的CARTS的心理测量特性，并和加拿大学生样本数据进行对比。

**方法：**79名帕杜瓦大学（意大利）的本科生完成了意大利语版的CARTS，以及对童年经历、精神健康、依恋的测量，并将其数据和288名加拿大学生使用英语完成CARTS进行对比。

**结果：**意大利语版本的内部一致性和与童年创伤问卷（Childhood Trauma Questionnaire）、父母联结测量工具（Parental Bonding Instrument ）的聚合效度达到可以接受的水平。在意大利样本中，相关分析发现关于依恋和虐待的CARTS母亲评分和浪漫依恋相关联，而CARTS父亲评分和PTSD症状与其它心理病理症状显著关联。意大利和加拿大学生在CARTS中关系类型有显著差异，意大利学生同时评估父母双方更少虐待性，同时也更少成为安全依恋源。

**结论：**这个初步研究的结果似乎支持了意大利语CARTS的聚合效度，以及其和童年依恋相关的经历、浪漫依恋之间的关联。研究发现了加拿大和意大利学生之间在依恋和虐待量表上的文化差异。未来的研究可以关注童年虐待关系情景的跨文化差异，以及意大利语CARTS的心理测量特性。

## 1. Introduction

The severe and long-lasting effects of childhood traumatic experiences on the biological and psychological development of victims have been routinely documented in the literature (e.g. Cicchetti & Toth, 2005; Teicher & Samson, 2016).

Among all the traumatic experiences that can be faced across life, children are minimally exposed to non-interpersonal trauma, such as car accidents, severe illness or natural disasters, compared to the incidence of interpersonal traumatic experiences of abuse, neglect and maltreatment (Van der Kolk & D’Andrea, 2010).

Studies of abused and maltreated children attest that victims rarely have experienced a single event, but rather they more often endure multiple types of interpersonal traumas, even across different developmental periods (e.g. infancy, childhood, adolescence). Indeed, children are much more likely to have undergone several traumatic experiences of an interpersonal nature and therefore different types of expositions (Greeson et al., 2011; Kisiel et al., 2014). Research has emphasized the difference between intra-familial and extra-familial traumatic experiences. Indeed, the importance of the relationship between parent and child is crucial for successful social and emotional development of the developing child (Schore & Schore, 2008; Sheeringa & Zeanah, 2001), and several studies have documented that individual differences in the interaction between parent and child are predictive of subsequent social and emotional adjustment (Apter-Levy, Feldman, Vakart, Ebstein, & Feldman, 2013; Bowlby, 1969; Schore, 2002; Simpson, Collins, Farrell, & Lee Raby, 2015), as well as of the child’s long-term ability to regulate intense emotions and stressful states (Van der Kolk, 2008, 2014). As a consequence, traumatic experiences of abuse and maltreatment within the family are likely to produce deeper effects, since they impact the attachment system and the caregiving function of providing the child with regulatory strategies for stress.

In order to outline trauma that generally is ‘environmental’ in nature, Schore (2001) defined the concept of ‘relational trauma’. Relational trauma represents the experiences of abuse, neglect and maltreatment embedded within existing attachment relationships and typically occurring within family; therefore, relational trauma is rarely a ‘single’ but rather a ‘cumulative’ event. When emotional maltreatment and neglect experiences occur between child and caregiver, the repetitive and prolonged unavailability and unresponsiveness of the caregiver produces a disruption of the attachment system. A prolonged, unrepaired and repetitively experienced attachment disruption becomes internalized as working model, shaping the child’s interpersonal expectations and behaviours, and increasing his/her vulnerability to psychopathology (Kobak, Zajac, & Madsen, 2016). In fact, severe threats to the availability of the caregiver, such as unrepaired attachment disruption, lead to persistent vulnerability, intense emotional reactions and defensive processes that compromise the relational functioning and contribute to psychopathological trajectories (Kobak, Zajac, & Madsen, 2016).

The emotional proximity between the victim and the abuser seems therefore to play a key role in the severity of relational traumatic experience. In addition, the complex emotional bond between the perpetrator and the victim makes trauma that occurs within the home more difficult to identify and more likely to be long-lasting (Goodman-Brown et al., 2003; Magalhães et al., 2009). In fact, intra-familial abuse and maltreatment are generally continuative, in comparison with over 62% of instances of extra-familial abuse being single case episodes. This evidence demonstrates the determining role played by the socio-ecological context on the duration and severity of child trauma as well as on the child’s response, evidencing that the more the attacker is close to the victim, the more the traumatic exposure is likely to last, and the more serious are likely to be the consequences (Kobak, Zajac, & Madsen, 2016).

Unfortunately, despite the fact that the literature is increasingly directing its attention on the abovementioned features defining the childhood trauma, up to now, the available assessment tools tend to overlook the socio-ecological and relational context of childhood trauma. For this reason, Frewen et al. (2013) developed the Childhood Attachment and Relational Trauma Screen (CARTS), an innovative assessment tool designed to measure instances of child abuse as well as warmth, security and support within the family, thus providing a socio-ecological relational perspective. This feature enables the evaluation of the subjective perception of the traumatic relational context, rather than only the frequency and severity of such experiences. The CARTS places in the theoretical framework of socioecology (Brofenbrenner, 1977; Belsky, 1980) and is based on the formulation of ‘relational trauma’ (Schore, 2002), assuming that childhood trauma occurs within the specific relationship between perpetrator and victims and that consequences of such experiences are largely conditioned by the relational and ecological context. Therefore, it uses a relationally contextualized survey methodology that asks what items apply as descriptions of the respondents’ family members and whether survey items apply as a description of him or herself, exploring the family dynamics of childhood relational trauma (Frewen et al., 2015a).

It is, therefore, important to emphasize that the CARTS not only takes into account the history and general severity of maltreatment and abuse but, more importantly, in which socio-ecological context maltreatment occurred (i.e. who did what and to whom). Moreover, the CARTS assesses thoughts, feelings and actions of the individual in response to their environment, in order to analyze the quality of early relationships and what role they played in the development of their home environment.

The existing research utilizing the CARTS has focused exclusively on English-speaking North American samples (Frewen et al., 2013, 2015a), demonstrating good psychometric properties in terms of reliability, convergent and predictive validity. So far, no cross-cultural studies have applied this promising tool beyond the North American population. However, cultural factors intervene on and across child development, suggesting multiple variations in parenting and child abuse. For example, ‘family values’ are strongly important within the Italian culture: Italians tend to encourage the development of strong family ties but also involve high expectations regarding obedience, discipline and compliance with parental rules. Such family duties are found to be among the main causes of conflict between parents and adolescents in Latin cultures. Instead, in the Canadian society, relationships between parents and children are described as in constant negotiation, during which the two sides are involved in the continuous redefinition of the relationship (Collins & Luebker, 1994; Liu et al., 2005). Moreover, Canadian parents tend to impose fewer limits and use inductive sanctions versus Italian parents who comparably tend to use punitive sanctions and infringe on children’s privacy. In addition, Hsu and Lavelli (2005) as well as Raudino and colleagues (2013) observed that, compared to American mothers, Italians show warm social and emotional behaviours as well as handling and holding; on the other hand, physical punishment is a discipline method quite accepted in Italy (Lansford, Alampay et al., 2010).

The current study thus evaluates the psychometric properties of an Italian translation and application of the CARTS in comparison to new data acquired from a Canadian sample. To extend the concurrent validity of the CARTS, we also investigated the association between retrospectively measured childhood attachment and abuse experiences (via the CARTS) and current adult romantic attachment and psychological symptoms in the Italian sample. Finally, we investigated whether response to the CARTS might be sufficiently sensitive to capture cultural variations between Italians and Canadians in parental attachment representations and relational trauma.

## 2. Method

### 2.1. Participants

The sample for the current study consisted of 85 Italian undergraduate students who completed the CARTS as well as other study measures, in comparison to 342 Canadian undergraduates. An inclusion criterion for the study was that participants describe both their biological mother and biological father on the CARTS. Therefore, 6 participants from the Italian sample and 54 participants from the Canadian sample were removed from subsequent analyses. The final sample (*N* = 79 Italian students, *N* = 288 Canadian students) was primarily female, and of young adult age, tending to be employed as students at the time of the study. See Table 1 for full demographic information of the sample.

**Table 1.** Sample demographics.

	Italian (N = 79)	Canadian (N = 288)
**Sex (%)**		
Female	69 (87.3)	182 (63.2)
**Age – M (SD)**	22.67 (1.42)	18.39 (1.32)
**Marital status (%)**		
Engaged	49 (62)	17 (5.9)
Single	26 (32.9)	262 (91)
Living together	3 (3.8)	3 (.09)
Married	1 (1.3)	6 (2)
Declined to answer	-	
**Education (%)**		
Undergraduate	8 (10.1)	165 (57.4)
Bachelor’s degree	71 (89.9)	
**Work (%)**		
Unemployed	72 (91.1)	149 (51.7)
Part-time/full-time job	7 (8.9)	139 (48.4)

### 2.2. Measures

#### 2.2.1. Childhood Attachment and Relational Trauma Screen (CARTS)

The CARTS (Frewen et al., 2013) is a computer-based self-report measure designed to assess overt instances of childhood maltreatment, as well as the general warmth, security and supportiveness of individuals within the respondents’ family and external environment. Item content also permits for certain items to be applicable to the respondent him/herself. The CARTS consists of verbal items along with visual stimuli of family members (i.e. graphics depicting members of the participant’s family as stick-figure representations); non-verbal (visual) stimuli have relevant roles in the modality of assessment since they may activate processing within the right hemisphere, thought to be dominant for attachment representations, whether secure or traumatic in nature (e.g. Schore, 2014). Participants are asked to type up to 11 people who ‘*were in your family when you were growing up*’ and for each select a label from a dropdown menu that defined their relationship to the respondent. Selection options were extensive and explicitly assessed the biological relationship of the respondent to each family member (e.g. ‘Biological Mother’ versus ‘Non-Biological Mother [e.g. adoptive, step-mother, etc.]’).

As described earlier, to be included in the present set of analyses, participants must have reported on both their biological parents. Nevertheless, the instructions given to participants allowed them to define ‘family’ as liberally as they wished such that extended family (e.g. grandparents, uncles, aunts and so on), friends and others (e.g. teachers) could be included, and specific family members (e.g. biological parents) could be excluded, entirely at the respondents’ discretion (Frewen et al., 2013).

Administration of the CARTS was fully conducted by computer; the complete CARTS procedure has been described in previous research (Frewen et al., 2013, 2015a). The Italian CARTS was translated from the original English version (Frewen et al., 2013) and utilized the same internet-based programming to administer it. Instructions, CARTS items and relationship labels were translated by two bilingual judges, following back translation procedure (Brislin, 1970). The final draft was revised by the last author who agreed on the final version of Italian CARTS.

Frewen et al. (2013) divided 69 CARTS items into: twelve items that measure positive relational bonds (Positive); eight items that assess attachment security (Secure); three items that evaluate negative emotions experienced by the respondent (Negative Affect); a single item intended to evaluate positive feelings (Positive Affect); four items that assess negative feelings in response to a behaviour from family members (Negative Feelings); five items that evaluate negative relational beliefs attributed to the respondent from other family members (Negative Beliefs From); five items that evaluate negative relational beliefs (Negative Beliefs To); two items that describe emotional abuse directed at the respondent and the respondents’ family, respectively (Emotional Abuse Self/Other); two items that describe physically abusive behaviour towards the respondent and the respondents’ family members, respectively (Physical Abuse Self/Other); six items that assess sexual abuse committed against the respondent (Sexual Abuse); and finally three items added to assess abusive events in a less explicit way (Bad Things). The complete list of Italian items is available in Supplementary Material; it should be noted that the construction of such items is consistent with a relational perspective on childhood maltreatment, in that items not only assess that the respondent  experienced and/or witnessed violence, but further request information regarding who was violent, and towards whom. CARTS scales evidenced good internal consistency reliability, though with some exceptions depending upon the type of family relationship rated (Frewen et al. [2013]). Previous studies of Frewen (Frewen et al., 2013;Frewen et al., 2015a) demonstrated the convergent validity of the CARTS through positive correlations with the CTQ (Bernstein & Fink, 1998) and with PTSD Checklist-5 (Weathers et al.,).

#### 2.2.2. Childhood Trauma Questionnaire-Short Form

The CTQ-SF (Bernstein et al., 2003) is a self-report instrument composed of 28 items that assess experiences of emotional, physical and sexual abuse, as well as experiences of emotional and physical neglect. Responses are made on a 5-point Likert scale from 1 (*Not true*) to 5 (*Very often true*). Twenty-five items relate to experiencing childhood abuse and indicate the severity of the experience, while three items assess for denial/underreporting. Previous research has demonstrated that the CTQ-SF has good reliability and validity (Bernstein et al., 2003; Hernandez et al., 2012; Thombs, Bernstein, Lobbestael, & Arntz, 2009). In the current study, we administered the Italian CTQ-SF (Sacchi, Vieno, Simonelli, 2017), in which the original five-factor structure is confirmed and the Cronbach’s alpha ranges across subscales between .87 and .96.

#### 2.2.3. Parental Bonding Instrument (PBI)

The PBI (Parker, Tupling, & Brown, 1979) is a self-report questionnaire that measures the respondents’ perception of caregiving behaviour received during childhood. The information is based on the subject’s memory about parents’ relationships during the first 16 years of age. The questionnaire consists of 50 items in total, with 25 items referring specifically to mother vs. father, respectively: 12 items explored parental care while the remaining 13 items measured overprotection/control. Scores are totaled on a 4-point Likert scale from 1 (*Absolutely true*) to 4 (*Never true*). Previous research has shown the PBI to be a reliable (α < .70) and valid assessment instrument (Parker, 1983; Parker et al., 1979; Picardi et al., 2013; Wilhelm, Niven, Parker, & Hadzi-Pavlovic, 2005).

#### 2.2.4. Experiences in Close Relationships-Revised

The ECR-R (Fraley, Waller, & Brennan, 2000) is a revised version of Brennan, Clark, and Shaver’s (1998) original ECR questionnaire. It consists of 18 items that measure anxiety and 18 items assessing avoidance (Sibley & Liu, 2004). The combination of these two scales determines the attachment style (secure: low anxiety, low avoidance; avoidant: low anxiety, high avoidance; anxious: high anxiety, low avoidance; fearful: high anxiety, high avoidance). The Italian version of the ECR-R has demonstrated good psychometric properties in terms of internal consistency (anxiety: α = .88; avoidance: α = .92), factorial and concurrent validity (Busonera, San Martini, & Zavattini, 2014; Calvo, 2008).

#### 2.2.5. PTSD Checklist for DSM-5 (PCL-5)

The PCL-5 is a 20-item self-report measure that assesses the 20 symptoms of PTSD based on DSM-5 criteria. It encompasses the previous versions of the PCL-4 (-C for civilian,-M for military and -S specific) and it reflects the changes proposed in the fifth version of the DSM for the diagnosis of PTSD. In the present study, the PCL-5 was administered without the Criterion A component. Scores are rated on a 5-point Likert scale from 0 (*Not at all*) to 4 (*Extremely*) based on the symptom severity experienced by the person over the past week . The PCL-5 has demonstrated good internal consistency (α = .96), test-retest reliability (r = .84) and convergent and discriminant validity (Armour et al., 2015; Bovin et al., 2016; Frewen, Brown, Steuwe, & Lanius, 2015b; Liu et al., 2014). In the present study, the internal consistency reliability resulted in .93.

#### 2.2.6. Symptom Checklist-90-R

The SCL-90-R (Derogatis, 1994) is a self-report questionnaire that assesses a wide range of psychological problems and symptoms. It consists of 90 items divided into nine subscales: Somatization (SOM); Obsessive-Compulsive (O-C); Interpersonal Sensitivity (I-S); Depression (DEP); Anxiety (ANX); Hostility (HOS); Phobic anxiety (PHOB); Paranoid ideation (PAR); and Psychoticism (PSY). Seven additional items (OTHER) explore disturbances in appetite and sleep. The Global Severity Index (GSI) is derived from the clinical scales and indicates the current level or depth of an individual’s distress or psychological disorder (Derogatis, 1994). Research from the Italian adaptation of SCL-90-R has found that all scales demonstrated acceptable internal consistency (α’s from .70–.90) (Prunas, Sarno, Preti, Madeddu, & Perugini, 2012).

### 2.3. Procedure

Italian and Canadian participants were undergraduate students recruited from the representing research institutions of the authors; they completed the CARTS and other measures surveying mental health and personality on private computers at a campus computer laboratory in the presence of an experimenter. The study protocol has been carried out in accordance with the recommendations of the Code of Ethics approved by the General Assembly of the Italian Association of Psychology.

## 3. Data analysis

Referring first only to results obtained with the Italian translation, psychometric characteristics of the Italian translation of the CARTS were undertaken replicating methods undertaken by Frewen et al. (2013). First, Kudar-Richardson-20 statistics were calculated for all CARTS subscales as measures of internal consistency reliability. Second, analysis of variance examined differential item applicability to four classes of response: Self; Biological Mother; Biological Father; and those that were determined to be Not Applicable to any person rated. Correlation analyses (t-b coefficients for ordinal-scales) were also performed between Mother and Father endorsement rates across CARTS scales. Third, convergent validity was evaluated in relation to CTQ (Emotional, Physical and Sexual Abuse, and Emotional Neglect subscales; Bernstein et al., 2003) and PBI (Overprotection and Care scales; Parker et al., 1979) subscales, utilizing multiple regression analyses. Finally, concurrent validity was studied in relation to adult psychopathology (SCL-90-R) and post-traumatic stress (PCL-5) symptoms, as well as for adult romantic attachment (ECR-R), applying Pearson’s correlation analyses.

We then examined cross-cultural differences between Italian and Canadian responses to CARTS scales and across the four relationship categories via split plot – ANOVA, with two within-subjects factors: Relationship (4 levels: Box; Self; Biological Mother; Biological Father), and Scale (CARTS subscales); and one between-subjects factor, Sample (2 levels: Italian vs. Canadian).

## 4. Results

### 4.1. Preliminary validation of the Italian CARTS

#### 4.1.1. Internal consistency of the CARTS Non-Applicable, Self, Mother and Father ratings

Descriptive statistics of the Italian translation of the CARTS are reported in Table 2. Overall, the Kudar-Richardson-20 coefficients indicated generally acceptable internal consistency for Non-Applicable, Self, Mother and Father ratings across CARTS subscales.

**Table 2.** Descriptive statistics and paired comparisons between Italian CARTS subscales ratings for Self, Mother and Father.

	Not Applicable^1^			Self^2^			Mother^3^			Father^4^			Correlations		
CARTS subscales	M	SD	α	M	SD	α	M	SD	α	M	SD	Α	Ʈ ^2–3^	Ʈ ^2–4^	Ʈ ^3–4^
Positive	.11	.35	.14	1.19	2.63	.92	9.4	3.75	.90	8.47	4.22	.91	.08	.20	.45**
Secure	.90	.82	.27				5.77	2.54	.85	3.89	3.19	.92			.29**
Negative Affect	1.36	.82	.42	.24	.62	.65	.36	.72	.59	.18	.49	.44	.15	.20	.13
Positive Affect	.64	.48		.24	.43			.50		.54	.50		.33**	.24	.46**
Negative Feelings From	3.43	1.72	.68				.55	1.22	.79	.34	1.01	.82			.29*
Emotional Abuse to Self	1.74	.57	.60				.11	.42	.85	.06	.29	.66			.37**
Emotional Abuse to Others	1.45	.66	.39	.00	.00		.08	.36	.80	.04	.26	.80			−.02
Negative Beliefs From	4.23	1.32	.79				.31	.86	.75	.23	.77	.78			.50**
Negative Beliefs To	4.4	1.22	.80				.12	.64	.89	.12	.53	.74			.70**
Physical Abuse to Self	1.92	.28	−.05				.05	.23	−.04	.06	.23	−.06			.38**
Physical Abuse to Others	1.92	.28	−.05	.14	.12		.04	.20	.00	.04	.21	−.04			−.03
Bad Things	2.89	.46	.74				.03	.16	.00	.03	.17	.00			.70**
Sexual Abuse	4.85	.83	.81				.01	.12		.01	.12	.00			1**

Notes: * *p *< .05; **p < .01, two-tailed

#### 4.1.2. Convergent validity of the CARTS

Table 3 reports the results of multiple regressions evaluating the convergent and concurrent predictive validity of the Italian translation of the CARTS. CARTS ratings in the scales of Emotional Abuse to Self, Physical Abuse to Self, and Sexual Abuse to Self, explained between 43% and 62% of the variance in CTQ subscale scores referring to Emotional, Physical and Sexual Abuse. In all cases, excepting convergence with CTQ Sexual Abuse, inclusion of CARTS parental ratings incrementally predicted additional variance in CTQ scores.

**Table 3.** Multiple regression analyses of Italian CARTS convergent validity with Childhood Trauma Questionnaire.

	Step 1	Step 2	Step 2	Step 2	Step 2
	Non-Applicable ratings	Mother & Father ratings	Non-Applicable ratings	Mother ratings	Father ratings
Dependent measure	R^2^	ΔR^2^	b(SE)	b(SE)	b(SE)
CTQ-EA	.38**	.08**	−2.06 (.65)**	2.01 (.76)**	2.01 (1.03)
CTQ-PA	.41**	.207**	−2.01 (.63)**	−.21 (.73)	4.14 (.70)**
CTQ-SA	.44**	.01	−1.28 (.18) **	−1.30 (.18)**	−1.30 (1.26)
CTQ-NE	.04	.39**	Positive: -.10 (1.12)	Positive: -.69 (.22)**	Positive: .16 (.19)
			Secure: .23 (.57)**	Secure: .08 (.25)	Secure: -.43 (.21)*

Notes: Step-1 predictors were CARTS Non-Applicability ratings, and Step-2 predictors were CARTS Mother and Father ratings. Dependent variables were CTQ-EA, CTQ-PA, CTQ-SA and CTQ-EN, CARTS subscale scores for Emotionally Abusive to Self, Physically Abusive to Self, Sexually Abusive to Self, and Positive and Secure were used, respectively. CTQ = Childhood Trauma Questionnaire- Short Form (Bernstein et al., 2003); EA = Emotional Abuse; PA = Physical Abuse; SA = Sexual Abuse; EN = Emotional Neglect. *p < 0.05, two-tailed, **p < 0.01, two-tailed.

Novel to the current study, compared with previous applications, was an examination of the convergent properties of the Italian translation of the CARTS relative to the CTQ Emotional Neglect subscale. We observed that 43% of the variance in CTQ Emotional Neglect was explained by CARTS ratings of Positive and Secure items, where 39% of the variance was explained by parental ratings beyond Non-Applicable ratings alone.

As regards the convergent validity of the Italian translation of the CARTS, ratings of Positive and Secure relationships relative to the PBI Care and Overprotection subscales, results demonstrated that CARTS ratings significantly predicted scores on the PBI-Care subscales (Table 4). In particular, CARTS ratings referring to Mothers predicted 53% of the variance in Mother Care; CARTS ratings referring to Father also predicted 53% of Father PBI-Care scores. Precisely, Mother Positive scores significantly predicted Mother Care score (b = .59, *p < *.01;), and Father Positive scores predicted father PBI-Care (b = .89, *p *< .01). Moreover, Security in Mother ratings predicted Mother PBI-Care (b = .86, *p *< .01), while Security in Father ratings showed no statistically significant effect (b = .44, *p *> .05) on Father PBI-Care. No statistically significant results (*p* > .05), were found for the PBI-Overprotection scale.

**Table 4.** Multiple regression analyses of Italian CARTS convergent validity with Parental Bonding Instrument.

	Step 1	Step 2	Step 2	Step 2	Step 2	Step 2
	Not- Applicable ratings	Mother & Father ratings	Non-Applicable Positive	*Non-Applicable Secure*	*Parent (M/F) Positive*	*Parent (M/F) Secure*
Dependent measure	R^2^	ΔR^2^	b (SE)	b (SE)	b (SE)	b (SE)
PBI-CARE (M)	.07	.46**	−.24 (1.34)	−.34 (.65)*	.59 (.20)**	.86 (.28)**
PBI-CARE (P)	.05	.48**	3.01 (1.74)	−.48 (.82)	.89 (.23)**	.44 (.32)
PBI-OVER (M)	.07	.03	−.99 (4.18)	1.91 (2.31)	.26 (.74)	−.76 (1.11)
PBI-OVER (P)	.03	.05	−.87 (2.13)	1.94 (1.01)	−.32 (.28)	.68 (.39)

Notes: Predictors were CARTS Positive and Secure subscale scores. Step-2 predictors of PBI-M were CARTS Mother ratings, whereas Step-2 predictors of PBI-F were CARTS Father ratings. PBI = Parental Bonding Instrument (PBI; Parker et al., 1979); PBI-CARE(M/F) = PBI-Care (Mother/Father); PBI-OVER (M/F) = PBI-Overprotection (Mother/Father). **p < 0.01, two-tailed

#### 4.1.3. Concurrent validity of the Italian CARTS

##### 4.1.3.1. Psychological symptoms

We also calculated correlations between CARTS subscales (for both Mothers and Fathers) and PCL-5 total scores and SCL-90-R total scores within the Italian sample (Table 5). Referring to Father ratings, the scales of Negative Affect, Negative Feelings From, and Negative Beliefs To, were positively correlated strongly with both PCL-5 and SCL-90-R scores. In addition, Emotional Abuse to Self and Negative Beliefs From significantly correlated with PTSD symptoms while Positive Affect displayed a negative correlation with general psychopathology. On the contrary, Mother ratings of Positive and Secure were negatively associated to general distress, while Physical Abuse to Other was positively associated to SCL-90-R. For PCL-5 scores, all comparisons were significant (i.e., *p*’s < .05) between Father and Mother ratings, referring to Negative Affect (*Fisher’s Z* = 2.42, *p *< .01), Negative Feelings From (*Fisher’s Z* = 1.97, *p *< .05), and Negative Beliefs To (*Fisher’s Z* = 2.10, *p *< .05). Similarly, for SCL-90-R scores, in all cases, comparisons were significant (i.e., *p*’s < .05) between Father and Mother ratings, referring to Negative Affect (*Fisher’s Z* = 1.94, *p *< .05), Negative Feelings From (*Fisher’s Z* = 1.72, *p *< .05) and Negative Beliefs To (*Fisher’s Z* = 1.98, *p *< .05).

**Table 5.** Bivariate correlations between Italian CARTS subscales for Mother and Father ratings, and PCL-5, SCL-90-R, ECR-R Anxiety and Avoidance scores.

	Mother ratings		Father ratings	
CARTS subscales	PCL-5	SCL-90-R	PCL-5	SCL-90-R
Positive	−.20	−.24**	−.13	−.17
Secure	−.18	−.25**	−.06	−.06
Negative Affect	−.01	−.07	.36**	.35**
Positive Affect	−.17	−.16	.21	−.28*
Negative Feelings From	.05	.02	.31**	.25*
Emotional Abuse Self	.02	−.07	.26*	.21
Emotional Abuse Other	.08	.05	.15	.07
Negative Beliefs From	.09	.06	.32**	.18
Negative Beliefs To	.13	.14	.31*	.31*
Physical Abuse Self	−.07	−.04	.16	.12
Physical Abuse Other	.20	.27*	.11	.09
Bad Things	.12	.17	.09	.22
Sexual Abuse	.19	.12	.20	.12
CARTS subscales	ECR-R Avoidance	ECR-Anxiety	ECR-R Avoidance	ECR-Anxiety
Positive	−.09	−.16	−.04	−.13
Secure	−.06	−.27**	−.07	−.08
Negative Affect	.06	.02	.14	.17
Positive Affect	−.19	−.29*	−.14	−.18
Negative Feelings From	−.02	.09	.06	.10
Emotional Abuse Self	−.13	−.04	.16	.17
Emotional Abuse Other	−.04	−.01	.07	.12
Negative Beliefs From	−.06	.01	.13	.22
Negative Beliefs To	−.06	−.03	.20	.22
Physical Abuse Self	−.03	.11	.04	.17
Physical Abuse Other	.13	.27*	.06	.20
Bad Things	.03	.03	−.04	.05
Sexual Abuse	.14	.08	.14	.08

Notes: PCL-5 = PTSD Checklist 5, SCL-90-R = Symptom Checklist 90 Revised; ECR-R = Experience in Close Relationship-Revised;* *p *< .05, ** *p *< .01

##### 4.1.3.2. Adult romantic attachment

Bivariate correlations were calculated between each CARTS scale and the Anxiety and Avoidance ratings from the ECR-R, separately for Mother and Father ratings, within the Italian sample (see Table 5). Overall, few correlations reached statistical significance referring to Mother ratings in the Anxiety scale, and were in the small-moderate range (*r*’s between −29 < *r *< .27)., while nothing statistically significant was found for Father ratings.

### 4.2. Evaluation of Italian CARTS ratings

Correlations of CARTS subscales across Mother and Father ratings are displayed in Table 2 for the Italian sample. Mother and Father ratings showed moderate to strong paired correlations for CARTS subscales of Positive, Secure, Positive Affect, Negative Feelings From, Negative Beliefs From, Negative Beliefs To, Emotional Abuse to Self, Physical Abuse to Self, Bad Things and Sexual Abuse. Only three subscales did not reveal statistically significant correlations.

Regarding the correlation between Self and Mother ratings, it was found that only the Positive Affect subscale was significantly associated, *r* (78) = .33, *p *< .01. No significant correlations were found between Father and Self ratings.

### 4.3. Cross-cultural comparisons

Greenhouse-Geisser corrected *F* statistics are reported, due to the violation of the assumption of sphericity in both within-subjects factors (Relationship labels, CARTS subscales). A significant main effect of Sample was found suggesting that there is substantial variation between Italian and Canadian students across the different relationship types for the different CARTS scales, *F* (1, 252) = 19.32, *p *< .001.

Further, each of the within subjects’ factors significantly interacted with the between-subjects factor: Relationship X Sample, *F* (2, 548) = 12.29, *p *< .001; Scale X Sample, *F* (2, 430) = 26.24, *p *< .001. Finally, a significant three-way interaction was identified, Relationship X Scale X Sample, *F* (6, 1481) = 5.37, *p *< .001.

Between sample multivariate effects were thus analysed at the univariate level specific to the Not Applicable, Self, Mother and Father ratings for each of the 13 CARTS subscales (see Table 6). In order to control for Type I error rate, p values have been adjusted with FDR (False Discovery Rate; Benjamini & Hochberg, 1995).

**Table 6.** Multivariate ANOVA (MANOVA) between Italian and Canadian sample referring to Box, Self, Mother and Father ratings.

	Italian (1)	Canadian (2)	Anova	Findings
CARTS Subscales	*M (SD)*	*M (SD)*	F	adjusted p-value^a^
BOX				*P*
Positive	.14 (.40)	.28 (.01)	1.35	.29
Secure	.95 (.74)	2.36 (2.03)	27.03**	.00
Negative Affect	1.45 (.75)	1.19 (.94)	3.72	.11
Positive Affect	.71 (.46)	.63 (.48)	1.23	.29
Negative Feelings From	3.43 (1.70)	1.89 (1.32)	53.54**	.00
Emotional Abuse Self	1.84 (.41)	1.23 (.81)	30.88**	.00
Emotional Abuse Other	1.51 (.60)	1.37 (.79)	1.78	.23
Negative Beliefs From	4.28 (1.23)	3.92 (1.55)	2.46	.17
Negative Beliefs To	5.57 (.86)	4.19 (1.50)	3.29	.11
Physical Abuse Self	1.94 (.22)	1.53 (.69)	21.06**	.00
Physical Abuse Other	1.97 (.18)	1.57 (.69)	18.65**	.00
Bad Things	2.88 (.50)	2.82 (.62)	.43	.52
Sexual Abuse	4.84 (.83)	5.73 (1.12)	31.88**	.00
SELF				
Positive	1.10 (2.60)	2.12 (3.33)	4.58	.15
Secure	-	-	-	-
Negative Affect	.19 (.54)	.17 (.47)	.09	.78
Positive Affect	.21 (.41)	.33 (.47)	3.31	.17
Negative Feelings From	-	-	-	-
Emotional Abuse Self	-	-	-	-
Emotional Abuse Other	00 (.00)	0.5 (.28)	1.89	.28
Negative Beliefs From	-	-	-	-
Negative Beliefs To	-	-	-	-
Physical Abuse Self	-	-	-	-
Physical Abuse Other	.00 (.00)	.03 (.22)	1.08	.38
Bad Things	-	-	-	-
Sexual Abuse	-	-	-	-
MOTHER				
Positive	10.02 (3.11)	11.3 (2.60)	9.93**	.01
Secure	3.67 (3.14)	7.21 (2.77)	9.73**	.01
Negative Affect	.29 (.62)	.42 (.76)	1.32	.33
Positive Affect	.53 (.50)	.69 (.46)	4.76	.08
Negative Feelings From	.40 (.97)	.63 (1.16)	1.99	.32
Emotional Abuse Self	.07 (.32)	.16 (.46)	1.88	.32
Emotional Abuse Other	.02 (.13)	.16 (.50)	4.83	.08
Negative Beliefs From	.24 (.68)	.18 (.68)	.32	.67
Negative Beliefs To	.03 (.15)	.26 (.69)	1.50	.32
Physical Abuse Self	.05 (.22)	.18 (.44)	4.88	.08
Physical Abuse Other	.03 (.18)	.09 (.34)	1.53	.32
Bad Things	.02 (.13)	.04 (.28)	.25	.67
Sexual Abuse	.02 (.13)	.04 (.28)	.10	.75
FATHER				
Positive	8.60 (4.00)	10.04 (3.10)	12.61**	.00
Secure	3.67 (3.14)	5.70 (3.10)	19.10**	.00
Negative Affect	.12 (.38)	.42 (.79)	7.95*	.03
Positive Affect	.57 (50)	.69 (.46)	2.88	.13
Negative Feelings From	.33 (.93)	.76 (1.27)	5.71*	.05
Emotional Abuse Self	.05 (.29)	.28 (.93)	3.29	.11
Emotional Abuse Other	.02 (.13)	.22 (.81)	3.59	.11
Negative Beliefs From	.19 (.66)	.19 (.52)	.00	.99
Negative Beliefs To	.10 (.55)	.20 (.55)	1.48	.30
Physical Abuse Self	.02 (.13)	.20 (.47)	8.79**	.00
Physical Abuse Other	.00 (.00)	.11 (.37)	4.84	.07
Bad Things	.02 (.13)	.05 (.35)	.53	.56
Sexual Abuse	.02 (.13)	.06 (.56)	.35	.60

Notes: *p < .05; **p < .01; ^a^p values adjusted with FDR (False Discovery Rate; Benjamini & Hochberg, 1995).

## 5. Discussion

Thanks to the increase in the globalization of psychology and its related fields, translating evidence-based assessment instruments developed in English into different languages is a very important responsibility (Van Widenfelt, Treffers, De Beurs, Siebelink, & Koudijs, 2005). In this regard, the literature points broadly out as parenting practices, including the use of physical punishment and other potentially abusive behaviours, take on different meanings depending on the cultural and social framework in which they occur (Sangawi, Adams, & Reissland, 2015). Consequently, the translation of existing assessment tools and their adaptation to the specificity of differing cultural contexts is necessary if childhood trauma is to be assessed in an evidence-based manner globally.

With few exceptions, the present preliminary results suggest acceptable internal validity of the CARTS subscales for the Italian translation. In particular, generally acceptable internal consistency has been proved, except for some values, where the very low endorsement rates for certain subscales (e.g. Physical Abuse to Self/Other and Sexual Abuse and Bad Things) resulted in low coefficients. Moreover, convergent validity was established, including being the first study to establish the correspondence of the CARTS with the CTQ Emotional Neglect subscale, and the PBI. Interestingly, we found that the CARTS item content appears to be independent of parental over-protectiveness as measured by the PBI, which measures such behaviours as ‘Tried to control everything I did’ and ‘Invaded my privacy’; this is in line with the fact that CARTS scales do not cover all dimensions of caregiving, such as overprotective behaviours.

Further novel to the current study was an assessment of the concurrent validity of the CARTS in relation to experiences of attachment in adult romantic relationships, where the literature suggests that experiences of childhood attachment predict future adult romantic attachment (Hesse & Main, 2000; Kochendorfer & Kerns, 2017; MacDonald et al., 2008). In the present study, select correlations between CARTS Mother ratings and responses to the ECR-R were significant, in particular in the association with concurrent anxious romantic attachment. Specifically, we found that the less secure and more negative Mothers were rated on the CARTS, the more likely that the individual struggled with anxiety in their current romantic relationships. Finally, CARTS ratings seem to be associated with general distress and PTSD symptoms. In this respect, it was interesting to observe that stronger associations tended to be found in relation to fathers’ ratings than mothers’ ratings among Italian students. That means that maternal ratings of attachment and abuse are more likely to show a continuity and thus shape the relational functioning in adulthood; on contrary, negative affect, feelings and internalized beliefs from the father appear to be less likely to impact interpersonal outcomes among Italian young adults, but rather have a punctual effect on PTSD symptoms, which include alterations in mood and cognition, such as negative feelings and affects.

Differences in mean ratings referring to mothers and fathers obtained from the Italian sample tended to replicate prior findings for mothers to be regarded as more positive and secure, yet there were no significant differences between parents regarding the perpetration of abuse.

More interesting, however, were the results of cross-cultural comparisons, where the current preliminary study seems to reveal several cultural differences in rating experiences pertaining to abuse and attachment between Italians and Canadians. First, referring to overall Non-Applicable Ratings, significant differences were found such that Italian students were more likely to identify persons within their childhood who were secure, were less likely to identify persons as having negative feelings from, and were less likely to identify persons who were emotionally abusive towards them, or physically abusive towards them or others. Despite this, however, Italian students were more likely to identify someone during their childhood history who was sexually abusive towards them. In contrast, Canadian students rated *themselves* as more often the source of positive feelings in comparison with Italian students, with a similar non-significant trend found for higher ratings of positive affect.

Further, referring to Biological Mother CARTS ratings, Canadian students rated their mothers as simultaneously significantly more often the source of positive and secure attachment. Finally, in reference to Father ratings, Canadian students likewise rated their fathers as more often the source of positive and secure attachment, yet also higher in negative affect, and more often the source of negative feelings.

Essentially opposite to what might be expected from prior literature (e.g. Lansford, Alampay, et al., 2010; Raudino et al., 2013), Italian students more often rated their mothers and fathers as less positive and secure, but less often the perpetrators of emotionally abusive behaviour toward them, as well as physically abusive behaviour both towards them directly and towards others in their family. Indeed, prior studies suggested that Canadian parents tend to be more diplomatic than Italians, who tend to enforce stricter rules on their children, sometimes also using corporal punishment, which remain more acceptable within the Italian culture as compared to North American families (Lansford, Alampay, et al., 2010; Lansford, Malone et al., 2010). In contrast to this, some studies (Hsu & Lavelli, 2005; Raudino et al., 2013) considered Italian mothers to be particularly warm, emotional and social caregivers. These variable findings might be linked to the influence exerted by cultural practices of a country on the parenting styles of its constituents (Claes, Lacourse, Bouchard, & Perucchini, 2003). In particular, what has generally been observed in the literature is that Canadian parents are more relaxed and less punitive than Italian parents. That means that the mild attitude of Canadian parents may lead young adults to consider their parents as more abusive when they react in stronger terms. Conversely, Italian young adults may only consider an abusive event significant if a parent is severely physically abusive (e.g. punching or kicking the child) or sexually abusive, whereas in Canada, instances of more mild physical punishment, such as spanking, are considered abusive in some contexts. Of course, this interpretation might be further supported by data on cross-cultural perceptions of potentially abusive behaviours’ severity and the current results pointing this direction should be corroborated by further studies on more representative samples. In any case the present findings do preliminarily suggest cultural variance exists between caregiving practices and that such variance can be captured in group responses to the CARTS, which may have implications for the development of distress and posttraumatic symptoms later in life.

The present findings must be evaluated in reference to some limitations of the research. First, the current study is conceived as a preliminary application of the CARTS out of North American context, and it proposes a first exploration of the psychometric properties of a translated version in the Italian context. As a consequence, the study had a relatively small sample size for definitive evaluation of the measure. In fact, concerning concurrent validity with romantic attachment and PTSD symptoms, the sample size of the Italian group might have impacted the significance of correlations, which appear in a low- to- moderate for Mother ratings especially. In addition, as regards the association between CARTS scales and romantic attachment, the sample size of the present study requires being careful in the interpretation of such results; further studies involving more participants might clarify the nature of the association. Also, further studies might involve the application of Structural Equation Modelling in exploring a predictive role of CARTS on romantic attachment. Overall, future studies with larger and more representative samples are needed to determine how these results can be considered generalized to populations that differ in gender distribution, socio-cultural features and family formation. Second, in the current sample a floor effect may have interacted with small sample size to lower reliability calculations (Warner, 2013), as most participants did not report any history of abuse, and certainly were unlikely to report severe occurrences of emotional, physical or sexual abuse. As a consequence, future cross-cultural research should apply the CARTS to different samples, such as clinical populations presenting with previously validated traumatic histories. Finally, this study is limited only to analyzing the responses that affect the respondent him or herself, his or her mother and his or her father; it could be informative for future researchers to evaluate the relations with other family members (e.g. siblings, grandparents and others) or even people outside the family (e.g. coaches, teachers and others) cross-culturally as was done with English participants previously (Frewen et al., 2015a).

We conclude that the current study preliminarily supports the internal consistency, concurrent and convergent validity of an Italian translation of the CARTS, through Cronbach’s alpha, regression and correlational analyses. In addition this study firstly introduces the CARTS to the subject of cross-cultural variations in relational attachment-based trauma. A larger study to investigate cross-cultural variation in the relational, socio-ecological context of childhood abuse, and in order to boost Italian CARTS psychometric features is warranted.

## Supplementary Material

Supplementary material
