# Supplementary material for: Italian translation and cross-cultural comparison with the Childhood Attachment and Relational Trauma Screen (CARTS)
Source: Eur J Psychotraumatol. 2017 Oct 10;8(1):1375839. doi: 10.1080/20008198.2017.1375839 (PMC5687796; doi:10.1080/20008198.2017.1375839)
Supplement: Supplementary material [file ZEPT_A_1375839_SM6592.docx]

| **Table 1.** Items listing of the Italian Childhood Attachment and Relational trauma Screen (Italian CARTS) | | | |
| --- | --- | --- | --- |
| 1. Mi piaceva molto questa persona. |  | Positive |  |
| 1. Volevo molto bene a questa persona. |  | Positive |  |
| 1. A questa persona piacevo molto. |  | Positive |  |
| 1. Questa persona mi voleva molto bene. |  | Positive |  |
| 1. Questa persona si prendeva cura di me. |  | Positive |  |
| 1. Questa persona ci teneva a me. |  | Positive |  |
| 1. Questa persona era orgogliosa di me. |  | Positive |  |
| 1. Questa persona mi dava baci e abbracci. |  | Positive |  |
| 1. Questa persona mi rendeva tranquillo/a. |  | Positive |  |
| 1. Questa persona mi rendeva felice. |  | Positive |  |
| 1. Questa persona mi faceva stare bene con me stesso/a. |  | Positive |  |
| 1. Mi divertivo molto con questa persona. |  | Positive |  |
| 1. Ero felice che questa persona facesse parte della mia famiglia. |  | Positive |  |
| 1. Mi rivolgevo a questa persona quando mi sentivo triste o turbato/a. |  | Secure |  |
| 1. Mi rivolgevo a questa persona quando mi sentivo impaurito/a o preoccupato/a. |  | Secure |  |
| 1. Mi rivolgevo a questa persona quando mi sentivo furioso/a o arrabbiato/a. |  | Secure |  |
| 1. Mi recavo da questa persona per ricevere aiuto se avevo un problema. |  | Secure |  |
| 1. Questa persona mi faceva sentire meglio quando ero triste o turbato/a. |  | Secure |  |
| 1. Questa persona mi faceva sentire meglio quando ero impaurito/a o preoccupato/a. |  | Secure |  |
| 1. Questa persona mi faceva sentire meglio quando ero furioso/a o arrabbiato/a. |  | Secure |  |
| 1. Questa persona mi aiutava quando avevo un problema. |  | Secure |  |
| 1. Questa persona era quasi sempre triste e turbato/a. |  | Neg. Affect |  |
| 1. Questa persona era quasi sempre furioso/a e arrabbiato/a. |  | Neg. Affect |  |
| 1. Questa persona era quasi sempre impaurito/a e preoccupato/a. |  | Neg. Affect |  |
| 1. Questa persona era solitamente felice. |  | Pos. Affect |  |
| 1. Questa persona mi faceva sentire triste e turbato/a. |  | Neg. Feel. From |  |
| 1. Questa persona mi faceva sentire impaurito/a e preoccupato/a. |  | Neg. Feel. From |  |
| 1. Questa persona mi faceva sentire furioso/a e arrabbiato/a. |  | Neg. Feel. From |  |
| 1. Questa persona mi faceva sentire male riguardo a me stesso/a. |  | Neg. Feel. From |  |
| 1. Questa persona mi chiamava con brutti nomi. |  | Emot. Abuse - Self |  |
| 1. Questa persona mi diceva cose davvero cattive. |  | Emot. Abuse – Self |  |
| 1. Questa persona chiamava le persone della mia famiglia con brutti nomi. |  | Emot. Abuse - Others |  |
| 1. Questa persona diceva cose davvero cattive alle persone della mia famiglia. |  | Emot. Abuse - Others |  |
| 1. Pensavo di non piacere molto a questa persona. |  | Neg. Beliefs From |  |
| 1. Pensavo che questa persona non mi voleva bene. |  | Neg. Beliefs From |  |
| 1. Pensavo che questa persona sperasse che io NON fossi parte della nostra famiglia. |  | Neg. Beliefs From |  |
| 1. Pensavo che questa persona pensasse che io fossi cattivo. |  | Neg. Beliefs From |  |
| 1. Pensavo che questa persona mi odiava. |  | Neg. Beliefs From |  |
| 1. NON mi piaceva molto questa persona. |  | Neg. Beliefs To |  |
| 1. NON volevo molto bene a questa persona. |  | Neg. Beliefs To |  |
| 1. Speravo che questa persona NON facesse parte della nostra famiglia. |  | Neg. Beliefs To |  |
| 1. Pensavo che questa persona fosse una cattiva persona. |  | Neg. Beliefs To |  |
| 1. Pensavo di odiare questa persona. |  | Neg. Beliefs To |  |
| 1. Questa persona mi schiaffeggiava, percuoteva o colpiva. |  | Phys. Ab. - Self |  |
| 1. Questa persona mi tirava pugni o mi prendeva a calci. |  | Phys. Ab. – Self |  |
| 1. Questa persona schiaffeggiava, percuoteva o colpiva persone della mia famiglia. |  | Phys. Ab. - Other |  |
| 1. Questa persona tirava pugni o prendeva a calci persone della mia famiglia. |  | Phys. Ab. – Other |  |
| 1. Ho assistito (guardato o sentito) questa persona essere minacciata o aggredita DA MIA MADRE |  | Wit-V by mom |  |
| 1. Ho assistito (guardato o sentito) questa persona essere minacciata o aggredita DA MIO PADRE |  | Wit-V by dad |  |
| 1. Ho assistito (guardato o sentito) questa persona essere minacciata o aggredita DA UNO O Più DEI MIEI FRATELLI |  | Wit-V by sibs |  |
| 1. Ho assistito (guardato o sentito) questa persona essere minacciata o aggredita DA UNA O Più DELLE MIE SORELLE |  | Wit-V by sibs |  |
| 1. Questa persona minacciava o aggrediva MIA MADRE |  | Wit-V to mom |  |
| 1. Questa persona minacciava o aggrediva MIO PADRE |  | Wit-V to dad |  |
| 1. Questa persona minacciava o aggrediva UNO O Più DEI MIEI FRATELLI |  | Wit-V to sibs |  |
| 1. Questa persona minacciava o aggrediva UNA O Più DELLE MIE SORELLE |  | Wit-V to sibs |  |
| 1. Questa persona mi faceva cose brutte che non avrei dovuto raccontare ad altre persone. |  | Bad Things |  |
| 1. Questa persona mi faceva fare cose brutte che non avrei dovuto raccontare ad altre persone. |  | Bad Things |  |
| 1. Questa persona mi faceva cose cattive di cui non mi piaceva parlare o pensare. |  | Bad Things |  |
| 1. Questa persona mi faceva toccare parti del suo corpo in posti dove non volevo lo facesse. |  | Sexual Abuse |  |
| 1. Questa persona toccava parti del mio corpo senza che io lo volessi. |  | Sexual Abuse |  |
| 1. Questa persona mi faceva toccare parti del suo corpo in posti dove non avrei dovuto. |  | Sexual Abuse |  |
| 1. Questa persona toccava parti del mio corpo dove non avrebbe dovuto. |  | Sexual Abuse |  |
| 1. Questa persona mi faceva fare cose senza i suoi vesti addosso. |  | Sexual Abuse |  |
| 1. Questa persona mi faceva fare cose senza i miei vesti addosso. |  | Sexual Abuse |  |
| 1. Mi piaceva molto questa persona. |  | Positive |  |
| 1. Volevo molto bene a questa persona. |  | Positive |  |
| 1. Piacevo molto a questa persona. |  | Positive |  |
| 1. Questa persona mi voleva molto bene. |  | Positive |  |
| 1. Ero felice che questa persona fosse della mia famiglia. |  | Positive |  |
